# Green Silver Nanoparticles Synthesized from *Taverniera couneifolia* Elicits Effective Anti-Diabetic Effect in Alloxan-Induced Diabetic Wistar Rats

**DOI:** 10.3390/nano12071035

**Published:** 2022-03-22

**Authors:** Muhammad Nisar Ul Haq, Ghulam Mujtaba Shah, Farid Menaa, Rahmat Ali Khan, Norah A. Althobaiti, Aishah E. Albalawi, Huda Mohammed Alkreathy

**Affiliations:** 1 Department of Botany, Hazara University, Mansehra 21300, Pakistan; mnhq1987@gmail.com (M.N.U.H.); gmujtabashah72@yahoo.com (G.M.S.); 2 Department of Internal Medicine and Nanomedicine, California Innovations Corporations, San Diego, CA 92037, USA; 3 Department of Biotechnology, University of Science and Technology, Bannu 28100, Pakistan; 4 Department of Biology, College of Science and Humanities, Shaqra University, Al Quwaiiyah 19257, Saudi Arabia; nalthobaiti@su.edu.sa; 5 Department of Biology, Faculty of Science, University of Tabuk, Tabuk 71491, Saudi Arabia; ae.albalawi@ut.edu.sa; 6 Department of Pharmacology, Faculty of Medicine, King Abdulaziz University, Jeddah 21589, Saudi Arabia; halkreathy@kau.edu.sa

**Keywords:** alloxan, animal model, AgNPs, green nanomedicine, *Taverniera couneifolia*

## Abstract

**Background:** Using a variety of chemical compounds and biomolecules, researchers have been working on new antidiabetic drugs for many years. Anti-diabetic research is increasingly using nanomaterials because of their unique qualities, such as their tiny size, biocompatibility, and ability to penetrate cell membranes for drug delivery. Using extract of *T. couneifolia* coated with silver nanoparticles as a model for diabetes mellitus research was one of the goals of this work. **Methods:** Uv-Vis spectroscopy was used to measure the TAgNPs surface plasmon resonance. FTIR spectroscopy confirmed the attached functional groups, XRD analysis confirmed the size and crystallinity, scanning electron microscopy revealed that the majority of the particles were spherical, and EDX performed the elemental analysis. For 21 days, alloxan-induced diabetic Wistar rats (N = 25, n = 5/group) were administered 10 mg/kg body weight of photosynthesized AgNPs as a standard animal model, while those in the untreated normal control group C, received distilled water as a control, diabetics who were treated with 0.5 mg/kg of body weight of glibenclamide, 10 mg/kg of methanolic *T. couneifolia* extract, and diabetics who were given 10 mg/kg of body weight of synthetic AgNPs derived from *T. couneifolia* in the DAgNPs group. At the conclusion of the treatment, lipid, liver and kidney profiles were re-examined to determine whether or not the treatment had been effective (day 21). Oral glucose doses of 2 g/kg of body weight were administered to each group, and blood glucose levels were measured at various intervals (day 21). Fasting glucose levels were measured using a glucometer. Each animal’s urine was tested for leukocytes, nitrites, and bilirubin using lab-made prepared assay kits. One-way ANOVA and Dunnett’s test were used for statistical analysis. **Results:** The surface plasmon resonance effect was examined with UV-vis, it showed a sharp peak at 412 nm. X-ray diffraction measurements indicated that the produced nanoparticles were between 15 to 31.44 nm in size. Alloxan-induced diabetic rats were fed AgNPs derived from phytosynthesized AgNPs, compared to diabetic control rats, diabetic rats treated with AgNPs showed a considerable improvement in their dyslipidemia status. Over the course of the days, it also lowered blood glucose levels. A reduction in blood glucose levels, a rise in body weight, and significant improvements in the lipid, liver, and renal profiles were also seen. **Conclusions:** The present findings revealed that plant mediated silver nanoparticles significantly improved the alloxan induced diabetic changes in various treated rats and might be used for the treatment of diabetes.

## 1. Introduction

Diabetes, which is defined by high blood glucose levels (BGL), is one of the most prevalent complications of changing human lifestyles, and is generally linked to dietary habits, age, and genetics. Patients with diabetes have an increased risk of cardiovascular illness, kidney disease, neurological diseases, and hypertension, according to data from the US Department of Health and Human Services [1]. Type I and Type II (diabetes mellitus) are the most common forms of diabetes, whereas gestationaldiabetes affects pregnant women. For the most part, Type 2 Diabetes (T2D) attracts the most attention since it affects a huge percentage of the world’s populous because to insulin resistance in adipose tissue, muscle and liver due to decreased insulin production from pancreatic β-cells [2].

Nanomedicine has been studied as a therapy for diabetes in the recent past [3,4]. For the treatment of diabetes, researchers are also developing a variety of anti-diabetic agents, however some are restricted by their poor pharmacokinetic qualities [4,5].

There are several factors that contribute to the development of diabetes but one of the most important is the oxidative stress caused by reactive oxygen species (ROS). When glucose and fatty acids are overloaded, ROS are formed [6]. This may be attributed to a variety of factors, including overeating and under-exercising. Apoptotic cell death in the pancreatic beta-cells, according to Rohdes, is the cause of insulin depletion in people with diabetes [7]. According to research by Volpe et al., oxidative stress may cause the death of pancreatic -cells and the subsequent occurrence of diabetes problems. Hyperglycemia causes diabetes-related issues because of an imbalance in ROS, which results in increased oxidative stress and cell death, according to them. It is so possible to manage these diabetic consequences by decreasing ROS production [8].

Because of the disease’s progressive nature requires a better treatment plan, including the development of new medications [9]. Diabetic patients require new preventative and treatment measures, since medication therapy has not been totally effective [10]. Despite the lack of safety and effectiveness, humans have long used several herbs to treat diabetes experimentally [7]. An alternative to synthetic agents must be tested now, which is why herbal medicine is being investigated [11].

Since the previous decade, the use of AgNPs in a wide range of industries has grown significantly. As an anti-microbial agent, silver was first employed in the 1990s as silver colloids to treat a variety of illnesses [12]. The size, distribution, and shape of AgNPs (agglomerates of nanoparticles) all influence their unique features [13]. Nanoparticles are attracting a lot of attention these days, thanks to their many potential uses in a variety of fields, including catalysis, optics, antimicrobials, and the manufacture of biomaterials [14,15]. To make AgNPs, a variety of methods have been used. In contrast to chemical, physical and microbiological synthesis, photosynthesis does not need a lengthy cultivation and maintenance procedure, dangerous chemicals, or significant energy requirements [16,17,18].

*Taverniera couneifolia* is a member of family Papilionaceae is 60–100 cm shrub with pubescent branches. Inflorescence uni-trifoliolate, with leaflets 0.6–2.5 cm long, whole, mucronate, pubescent (at first), then subglabrous; stipules connate, and amplexicaul (at first), about three millimetres long. Axillary raceme up to 10 cm long inflorescence, bracts c. 2.5 mm, pedicel 1–2.5 mm long, Calyx 4–5 mm length, silky, 2.5 mm long teeth deltoid, purple with macrescent flecks on the corolla. The vexillum and keel are both larger than the wing’s span. Vegetables having 1–3 echinate and ovoid joints, pubescent joints [19]. The present project was therfore arranged to investigate the antidiabetic potency of biogenic nanoparticles prepared by *T. couneifolia* methanol extract using rats as an animal model.

## 2. Materials and Methods

### 2.1. Chemicals and Instruments

Aqua regia, DMSO, AgNO_3_, NaOH, ddH_2_O, formalin, normal saline solution, and diethyl ether were among the chemicals, disposable syringes, a dissecting board, cotton and a box contains surgical instruments were used.

Various instruments including Shimadzu UV-1800 UV-vis spec-trophotometer, FT-IR (PerkinElmer model-983/G detector, Chelmsford, MA, USA), SEM (JSM, JEOL 3010, Tokyo, Japan), XRD (JDX 3532 JEOL, Tokyo, Japan) were used in the current project.

### 2.2. Plant Collection and Extraction

*T. couneifolia* was collected in its full matured form during the month of May 2019 from the area of Barganato federally administered areas (FR), area of district Bannu, Khyberpakhtoonkhwa Province, Pakistan.

After being cleaned three times in distilled water, it was placed in a cool, dry place to dry for fifteen days before being used. The plant was ground into a fine powder. This was followed by vigorously shaking 100 g of powder in 300 mL of 75 percent methanol. The methanol plant extract was dried in a rotary evaporator and filtered via Whatmann Grade 1 filter paper after seven days. Weighted and kept at 4 °C, the resultant thick sticky gummy crude extract will be used in future research [20].

### 2.3. Investiagtions of Phytoconstituents

Various Bioactive plant metabolites were checked according to previously published protocols [21,22]. Briefly, the following tests have been used for the detection of phytochemicals:

*Test for alkaloids:* The ammonia solution (3 mL) was added to a test tube containing 1 g of dry powder samples. For a short period of time, they were permitted to stand. To remove the powder samples, 10 mL of chloroform was added to the test tube samples, shaken, and then filtered. Using a water bath, chloroform was evaporated, and 2 mL of Mayer’s reagent was added to the mixture. The existence of a cream-colored precipitate confirms the presence of alkaloids.

*Test for glycosides:* The extract is degraded in tab contains water for two hrs with concentrated HCl before being filtered. When the chloroform layer has been separated, 10 percent ammonia solution is added to the chloroform layer and shaken into the filtered hydrolysate, which now contains 2 mL of hydrolysate. Glycosides are indicated by a pink color.

*Test for saponins*: *T. couneifolia* crude extracts were diluted with dis-tilled water (20 mL) and the test tube was shaken by hand for 15 min to remove the stock solution. No foam formation indicates absence of Saponins.

*Test for proteins:* To treat the filtrate, a single drop of a 2 percent copper sulphate solution is added to a 2 mL solution. Ethanol (95 percent) and potassium hydroxide pellets are added to this mixture. The presence of proteins is shown by the ethanolic layer becoming pink.

*Test for amino acid:* Naphthalene solution (10 mg ninhydrin in 200 mL acetone) is added to 2 mL aqueous filtrate with two drops of ninhydrin solution. There was no color change observed conforms the absence of amino acids.

*Test for phytosterols:* Acetic anhydride is used to dissolve the extract (50 mg). A further drop or two of strong sulfuric acid is added to this mixture. The absence of phytosterols was indicated by no color change.

*Test for phenolic compounds:* Using 50 mg of extract, 5 mL of distilled water has been used to make the solution. Add a few drops of a neutral 5 percent ferric chloride solution to stabilize it. The presence of phenolic compounds is indicated by a dark green color.

*Test for flavonoids*: The stock solution of *T. couneifolia* was diluted with sodium hydroxide solution and a few drops were added (0.5 mL). The plant crude extract had a bright yellow colour before the addition of a few drops of diluted H_2_SO_4_ acid, which made it colourless. This indicates that flavonoids are present.

*Test for tannins:* The stock crude extract solution (0.5 mL) was dissolved in chloroform (5 mL) and added acetic anhydride (1 mL). Finally sulphuric acid (1 mL) was added carefully to the solution along the wall sides of the vessel. A green colour was formed, showing the presence of tannins.

*Test for coumarins:* 1 mL of extract was mixed with 1 mL of 10% NaOH. Coumarins may be detected by the yellow color they produce.

### 2.4. Green Synthesis of AgNPs

As previously reported, the bottom-up technique was used to synthesize AgNPs. AgNO_3_ (0.085 mg) was dissolved in 500 mL of ddH_2_O to prepare 1 mM solution, The crude extract (100 mg) was mixed with 50 mL of double-distilled water and before a specific amount of extract was obtained (100 µL, 300 µL, 500 µL, 800 µL, 1000 µL, 1500 µL, 2000 µL, 2500 and 3000 µL. 10 mL for each sample). An ultraviolet range of 200–800 nm was fixed. The pH was varied between 6 and 12. At pH 11 a sharp peak at 412 nm, was obtained which indicates the formation of AgNPs.

### 2.5. Physicochemical Characterizations of AgNPs

Various routinely used techniques (i.e., FTIR, XRD, UV-Vis, EDX and SEM were used to characterize the freshly synthesized AgNPs [23].

UV-Vis spectroscopy in the wavelength range of 200–800 nM was used to evaluate the surface plasma resonance (SPR) bonds of the biosynthesized AgNPs, in accordance with a previously described approach, their surface composition of active/functional groups were carried through FTIR spectroscopy within the mid-range of IR 4000–500 cm^−1^, according to a previous published method. Their size and crystal structure were investigated by XRD, according to a method previously reported. Their morphology was observed by SEM, following a previous method. Their elemental composition of photosynthesized AgNPs was analyzed by EDX [24].

### 2.6. In Vivo Induction of Diabetes

The method of Sengottaiyan et al. [25] was followed. Alloxan 200 mg per kg, intrapertoneally was injected into albino Wistar rats, aged eight weeks and weighing 140–150 g resulted in diabetes mellitus in the animals (DM). After 72 h, those with blood glucose levels of 220 mg/dL or above were diagnosed with diabetes.

### 2.7. In-Vivo Acute Toxicity

The acute toxicity of AgNPs was determined by administering dosages of 10 or 20 mg per kg of body weight. For up to 72 h, a trained (in the presence of a physician) observer maintained note of any neuro-logical, physiological, and behavioural changes in the animals, recording any deaths or lethality on a daily basis. As previously described [25], the experiment was conducted out with 10 mg/kg body weight AgNPs.

### 2.8. Experimental Treatments in Animals and Ethics

In Islamabad, Pakistan, the National Institute of Health (NIH) Sciences bought the albino Wistar rates. The NIH suggested pellets of feed were provided to them.

Furthermore another step to split the animals (n = 25) into 5 groups with each group consisting of 5 animals.

C: Normal control got just distilled water.

DAC: diabetic control, alloxan 200 mg/kg body weight.

DG: Glibenclamide 0.5 mg/kg body weight administered to diabetes rates.

DE: *T. couneifolia* extract 10 mg/kg body weight administered to diabetes rates.

DAgNPs: Diabetic rates that received 10 mg/kg of *T. couneifolia*-derived AgNPs.

After 21 days the animals were sacrificed through diethyl ether anesthesia.

### 2.9. Oral Glucose Tolerance Test

Each group was given 2 g/kg body weight of glucose and the blood glucose level was checked every 30 min for 30 min, 60 min, 90 min, and 120 min after the glucose was administered in order to perform an oral glucose tolerance test. Tests for fasting blood sugar levels were also carried.

### 2.10. Serology of Various Treated Groups

Via the retro-orbital puncture blood samples were taken and at 1000× *g* for 15 min samples were centrifuged. The serum was collected and kept at low temperatures (−80 °C). The results were then analyzed. The LDL-c, HDL-c, triglyceride levels, total cholesterol, total protein, bilirubin, creatinine, and ALT and ALP levels were determined using the method [25,26,27].

### 2.11. Urine Analysis

Samples were taken from each animal and varied levels of leukocytes, nitrite and urobilinogen were measured as well as leukocytes, nitrite, urobilinogen and protein.

### 2.12. Statistical Analysis

All the data were expressed as mean ± SD from three independent experiments, and the statistical significance between the groups was analyzed using one-way ANOVA followed by Tuckey test using Origin 18. If *p*-value ≤ 0.05, the data were considered significant.

## 3. Results

### 3.1. Qualitative Assesment of Bioactive Metabolites

The crude methanolic extract of *T. couneifolia* included terpenoids, quinons, carbohydrates, phenols, alkaloids, flavonoids, tannins, glycosides and amino acids, the crude extract did not exhibit any colour change for coumarins, sterols, proteins and saponins (Table 1).

### 3.2. Green Synthesis of AgNPs

**Figure 1:**
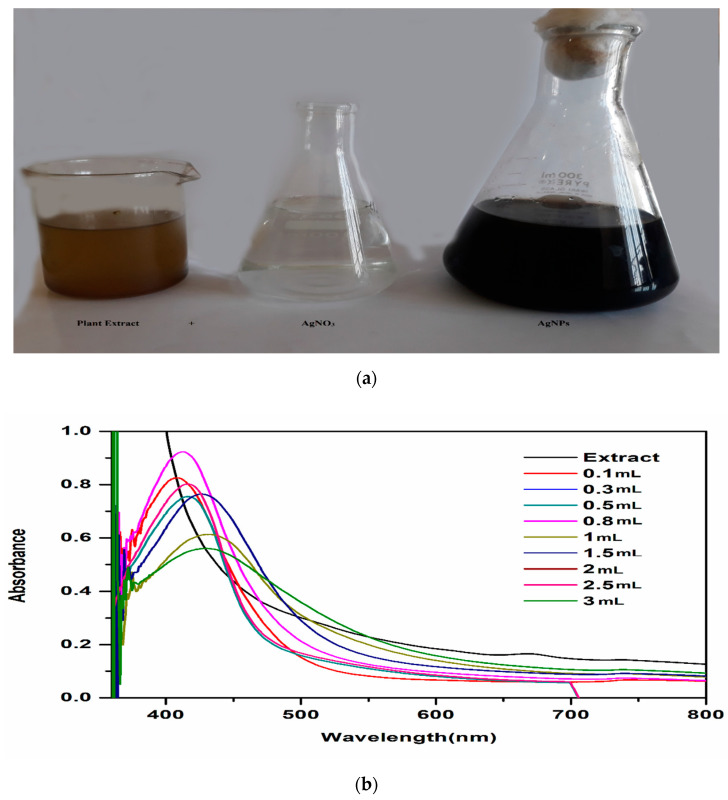
UV spectrum of biosynthesis of silver nanoparticles (**a**,**b**).

*T. couneifolia* extract (100 µL, 300 µL, 500 µL, 800 µL, 1000 µL, 1500 µL, 2000 µL, 2500 µL and 3000 µL) and a silver nitrate solution (0.085 g by weight) 1 mM solution were combined and the colour of the solution changed from yellow to dark brown, indicating the reduction of silver ions (Ag+) and the formation of nanoparticles (Figure 1a). The synthesis mechanism was explained by the SPR effect. There are a lot of flavonoids, sugars, phenolic constituents, and alkaloids in the *T. couneifolia* plant, which helped to form AgNPs.

### 3.3. Physicochemical Characteristics

#### 3.3.1. UV-Vis Analysis

At 412 nm and 0.5 to 0.9 intensity, the sharpest absorbance peak was recorded after 24 h, regardless of the quantity of AgNPs (Figure 1b). The various sizes of AgNPs were attributed for the wavelength discrepancy.

#### 3.3.2. FTIR Analysis

**Figure 2:**
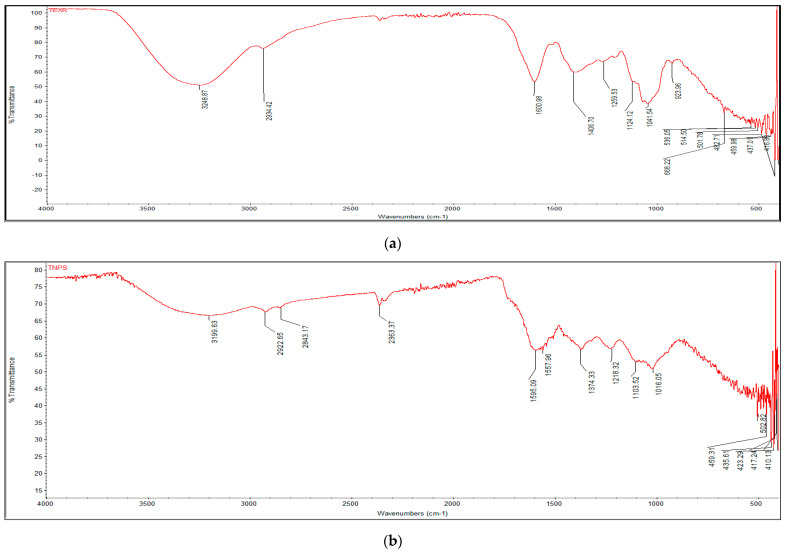
(**a**) *T. couneifolia* methanol extracts FTIR spectra; (**b**) Biogenic NPs FTIR Spectra.

*T. couneifolia* plant extract functional groups were identified and predicted by FTIR measurements. For both plant extract and AgNPs FTIR spectra were recorded (Figure 2). The FTIR signal at 3248 cm^−1^ was ascribed to O-H stretching and bending bonds in the crude plant extract. Phenols are clearly visible in the sample because of the prominent peak in the O-H band. As a result of the peak at 2934 cm^−1^ (meth-ylene stretching), symmetric and nonsymmetric C-H stretching bands have been created. The C=C stretch at 1600 cm^−1^ shows the conjugation of two aromatic rings of phenols. The peak at 923 cm^−1^ is not in the plan C-H bend and vibration. IR absorption tests reveal the presence of flavonoids, also known as aromatic cyclic phenols, in plant extracts from this area. The IR spectra of the produced AgNPs clearly show that plant biomolecules interacted with AgNPs, most likely via their oxygen functions, which serve as reducing and capping agents.

#### 3.3.3. XRD Analysis

**Figure 3:**
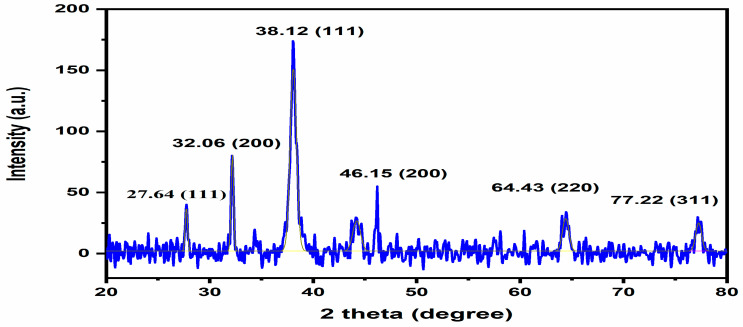
XRD pattern of *T. couneifolia* AgNPs.

Bragg reflections with twotheta degree values of 27.64°, 32°, 38°, 46°, and 64° match the XRD pattern’s (111), (200), (111), (220), and (311) sets of planes, respectively. This shows that the phytogenic AgNPs are crystallised (Figure 3).

Based on these findings, we may conclude that AgNPs have a face-centered cubic structure. This structure’s lattice constant was determined to be ¼ 4.0855 Å (Miller equation), and different sized AgNPs such as (31.22, 31.41, 11.90, 10.35, 13.65, and 15 nm) and its average size was determined 18 nm using the Scherrer equation.

#### 3.3.4. SEM Analysis

**Figure 4:**
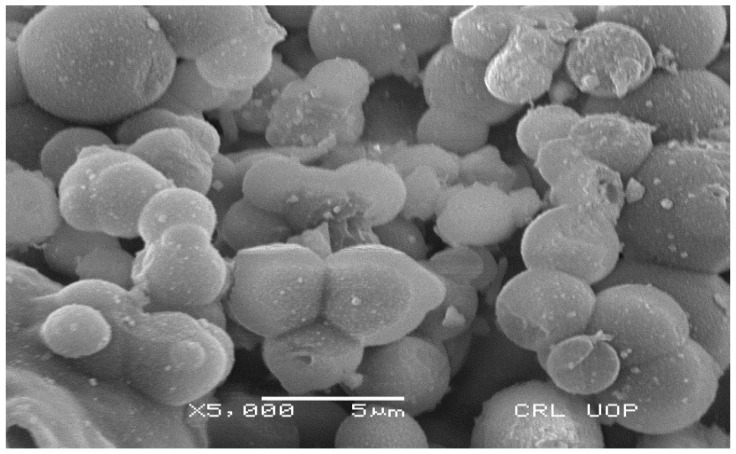
SEM image of *T. couneifolia* AgNPs.

SEM examination of powdered AgNPs was used to identify the morphology of the phytogenic AgNPs. Even after seven days, the green AgNPs maintained their spherical form and did not aggregate (Figure 4).

#### 3.3.5. EDX Analysis

**Figure 5:**
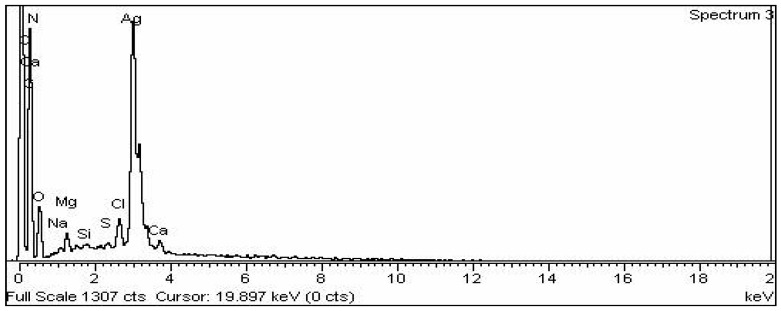
EDX spectrum representing elemental analysis of the greenly synthesized AgNPs.

The EDX detector linked to the SEM machine was used to determine the elemental composition of the biosynthesized AgNPs (Figure 5). In the graph, a sharp peak at 3 Kev proved that Ag was present, supporting the existence of AgNPs. 41.19 percent Ag was present of the total makeup. As well as C and N and O and Mg and Si and Cl were discovered using EDX investigation.

### 3.4. In Vivo Effects of T. couneifolia-Mediated AgNPs on Diabetes

#### 3.4.1. Phytogenic AgNPs Does Not Cause Acute Toxicity

The potential toxicity of the phytogenic AgNPs was checked for any neurological and behavioral changes. AgNPs were administered (orally) in rats using different doses (10–20 mg/kg) and were checked at various time intervals (i.e., 2, 24, 48, and 72 h). No changes were found from the phytogenic AgNPs. Thus, 10 mg/kg dose was selected as safe for our subsequent experiments.

#### 3.4.2. Phytogenic AgNPs Lower High Blood Glucose Levels

Alloxan-induced diabetes rates (DG, DE, and DAgNPs groups) were compared to Glibenclamide (0.5 mg/kg), which was utilized as a reference medication, at various time intervals (i.e., beginning day/day 1, day 7, day 15, and day 21).

**Figure 6:**
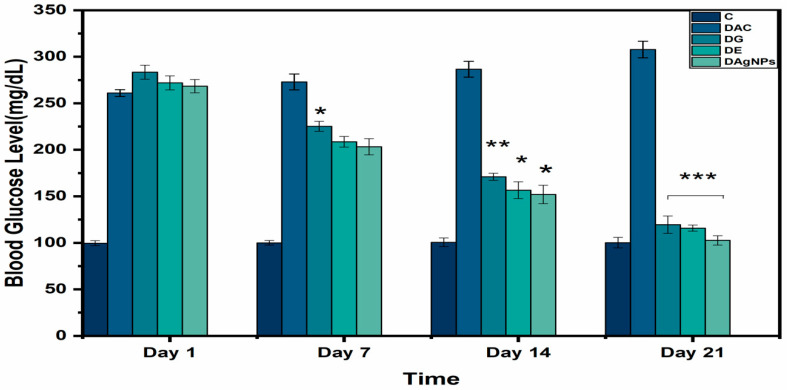
Blood glucose analysis of various treated groups; * *p* < 0.05; ** *p* < 0.01; *** *p* < 0.001 reveals statistically difference between groups.

The group DG is greater than D. AgNPs and DE is greater than T. AgNPs in terms of blood glucose level improvement (Figure 6).

**Figure 7:**
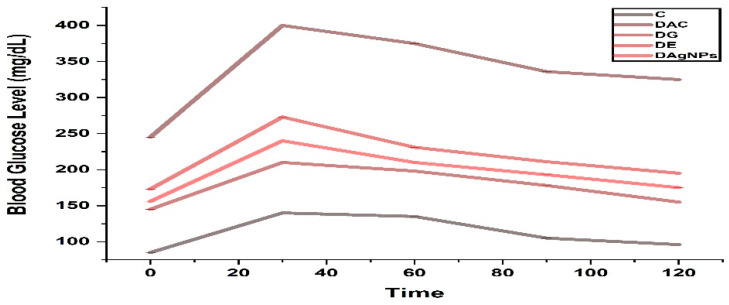
Effect of methanolic extract and synthesized *T. couneifolia* AgNPs on oral glucose tolerance test.

A glucose tolerance test was used to evaluate the improvement in hyperglycemic conditions among groups of rats treated with 10 mg/kg AgNPs. Glucose was administered orally to the animals. Glibenclamide (0.5 mg/kg body weight) was utilized as a reference medication to evaluate the impact of extract and AgNPs on Glibenclamide’s activity (Figure 7). Animals with diabetes that were treated with AgNPs (DAgNPs) exhibited a substantial drop in glucose levels compared to those who were not treated. Glycemic levels declined in a similar fashion during a period of 120 min: Greater than DAgNPs and DE is DG (the group DG was administrated with standard drug glibenclamide). As a result, compared to DG, phytogenic AgNPs reduced blood glucose levels in alloxan-induced diabetic rats.

**Figure 8:**
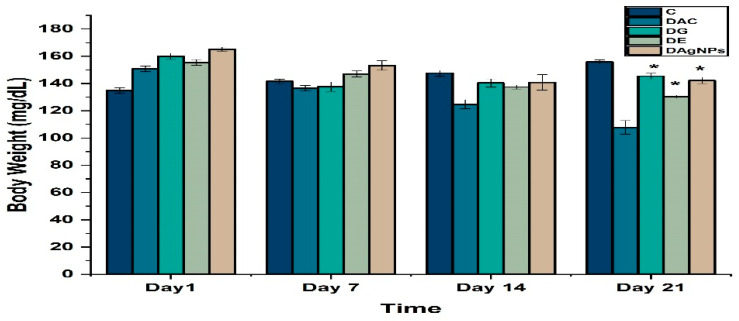
Body of various treated groups; * *p* < 0.05 reveals statistically difference between groups.

Alloxan induced diabetes effected the body weight of various treated groups. The results showed loss of body weight in diabetic animals (DAC group) as compared to that of control (C group) (Figure 8). Rats treated with NPs and extract markedly improved the changes in body weight.

#### 3.4.3. Serological and Urine Parameters

**Figure 9:**
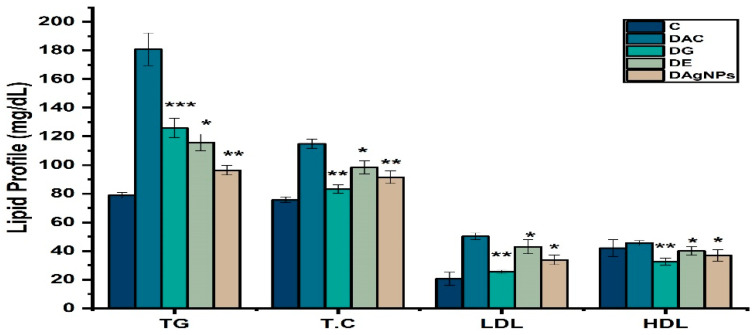
Lipid profile of various treated groups; * *p* < 0.05; ** *p* < 0.01; *** *p* < 0.001 reveals statistically difference between groups.

Blood cholesterol and lipid profile play a crucial role in the metabolism. Diabetes is being a metabolic disorder is affected in the experiment by the various treatments. Result showed levels of cholesterol, HDL-c, LDL-c, and TGs were increased in DAC group rats as compared to that of DC (Figure 9). However significant recovery effects were demonstrated by DAgNPs and DE groups.

**Figure 10:**
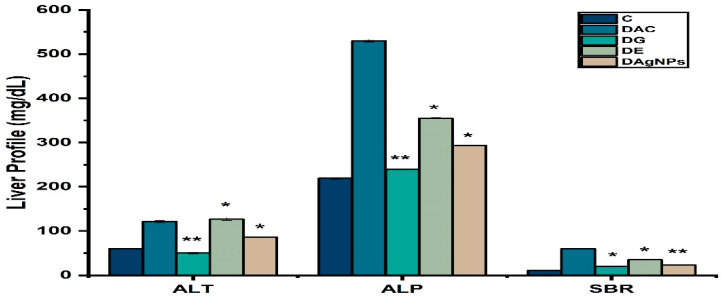
Liver profile of various treated groups; * *p* < 0.05; ** *p* < 0.01 reveals statistically difference between groups.

Liver profile is very sensitive in diabetes. In the present study alloxan caused diabetes caused abnormal changes in liver profile of various treated rats. Finding showed tha ALT, ALP, and SBR levels in DAC were higher than in C, indicating impaired liver function (Figure 10). The abnormal changes were restored by the treatment of by DAgNPs and DE in rats.

**Figure 11:**
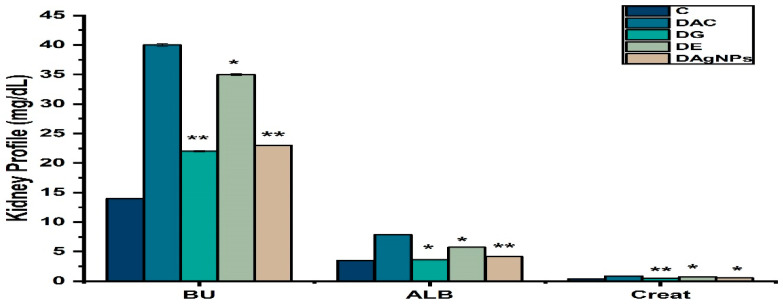
Kidney profile of various treated groups; * *p* < 0.05; ** *p* < 0.01 reveals statistically difference between groups.

Diabetes and hyperglycemia also showed abnormal serological effects in kidney of various treated groups. Finding showed that DAC animals had higher blood urea, albumin protein and creatine levels than C animals (Figure 11).

Urine analysis plays a crucial role in the anti-diabetic activities of silver nanoparticles. Diabetes mellitus in addition to serum abnormality also affects the urine profile of various treated rats. Urine analysis of the present project confirmed that most of the urine constituents are increased in diabetic animals compared to that of control animals (Table 2).

## 4. Discussion

It is well-known that phytochemical components are physiologically active chemicals, and that they exert their effects on a wide range of health conditions via a variety of biological pathways [28].

The polar plant crude extracts included the majority of secondary metabolites [29]. After biochemical screening, methanolic extracts of *T. couneifolia* included phenols, proteins, tannins, alkaloids, sterols and polysaccharides, amino acids, and terpenoids. It is possible that these bioactive chemicals are responsible for their antibacterial and antioxidative properties. The flavonoid groups that have shown high potential biological activities, such as antioxidant, anti-inflammatory, antibacterial, anti-cancer, and anti-allergic responses, are well documented [30,31,32,33]. Phenomenal compounds known as tannins and its derivatives are regarded as main antioxidants or free-radical scavengers, respectively [34,35,36,37,38].

For the synthesis of NPs, diverse portions of plant extracts are environmentally benign, cost-effective, and safe. A methanolic extract of the whole *T. couneifolia* was used to make AgNPs in the current work.

In order to synthesis AgNPs, the addition of extract and the pH value were optimized.

It was necessary to fine-tune these two variables in order to produce AgNPs with the desired properties.The colorless solution became brown, suggesting the development of silver nanoparticles (AgNPs) in it. Spectroscopic detection of AgNPs was made easier by the brown hue of silver, which is well-known. As with other plant extracts, AgNP synthesis might take anywhere from a few minutes to many hours [39]. The flavonoids and polyphenols in Camellia sinensis were shown to be nanoparticle capping agents in the research [40]. Hudlikara et al. [41] synthesized titanium dioxide NPs in the 25–100 nm range using *Jatropha curcas* L. as a capping agent together with curcacycline A and curcacycline B [42]. With the 4–30 nm wavelength, Ocimum sanctum leaf extract yielded Ag NPs in only 8 min. The proteins serve as a protective covering for the nucleic acids. S. aureus and E. coli may be suppressed [43,44]. Silver nanoparticles 10 nm in size was found in 25 min at 45 °C, while using the plant *Solanum xanthocarpum* [45]. Plants, bacteria, and other life forms, as well as bio-molecules, are often utilized in the production of NPs. Non-toxic or less dangerous compounds are used in this kind of synthesis.

When aqueous silver nitrate solution was added to geranium leaf broth (*P. graveolens*), stable crystalline silver nanoparticles were synthesized extracellularly by enzymes. The bioreduction of metal ions in solution resulted in a significant concentration of stable silver nanoparticles in the 16–40 nm size range. Open, quasi-linear superstructures of synthesized nanoparticles were seen, and the majority of them seemed to be cylindrical in shape [46].

With increasing exposure to time, UV spectra indicated a high absorption peak at 411 nM. *T. couneifolia* extract contains phytoconstituents that may reduce the quantity of silver ions (Ag+) in the aqueous solution, which might explain the rise in intensity. Similar outcomes were shown to be true [47,48].

FTIR has become a significant method for determining the functional groups involved in the interactions between metal particles and biomolecules. Chemical analysis of the AgNPs’ surface composition is carried out using this technique, as are searches for biomolecular capping and stabilizing agents. Ag+ ions may have been reduced by the presence of several functional groups. A potent reducing agent, like the flavonoids in the *T. couneifolia* extract, may indicate the synthesis of AgNPs via the reduction process. It is yet unknown how it works, therefore further research is needed [49].

T AgNPs were found to be crystallized by XRD at a size of 18.92 nm. Uv-vis spectra from the liquid after 25 days showed the same findings.

*T. couneifolia*-mediated AgNPs were found to be spherical, hexagonal, and irregular in form, as determined by SEM examination. Plants and plant components have different NP shapes and sizes. Phytoconstituents such alkaloids, flavonoids, tannins, and phenols were present since the whole plant was employed in this research. Our findings are in accord with those of earlier research. Squamosal leaf extract was discovered to contain AgNP particles with an average diameter of between 20 and 100 nM, according to the study [49]. *Desmodium gangeticum* AgNPs, on the other hand, showed spherical nanoparticles of 8 to 90 nM in diameter [33]. *T. couneifolia* reduced silver ion to elemental silver, as seen by the sharp silver signal peak. Metallic AgNPs generally exhibit an optical absorption peak of around 3.25 keV as a consequence of the SPR process.

From EDX spectrum, it was clear that *T. couneifolia* had percent yield of 41.57% of AgNPs, and this is also in agreement with previous studies [50].

Acute oral poisoning of AgNPs usually results in animals going through a series of behavioral abnormalities. Observations were taken over a period of time ranging from 0 min to 72 h in order to determine whether or not the test medications caused any undesirable or harmful side effects in the animals. This study’s findings demonstrate that the medications in question did not cause any negative side effects in the animals that were tested. Phytogenic AgNPs have been studied before [51,52,53,54,55].

There was no difference in blood glucose levels between normal control and diabetic rats (alloxan-induced diabetic rats) [56]. Uncontrolled glucose management in diabetic rats has resulted in hyperglycemic conditions, which have been seen [57,58]. The fact that diabetic rats treated with AgNPs return to normal levels in terms of biochemical indicators shows that AgNPs are helpful. *T. couneifolia* extract had less hypoglycemic effect than AgNPs in alloxan-induced diabetic rats. *T. couneifolia* extract had no effect on blood glucose levels after 21 days of oral administration. When given glucose orally, diabetic rats were tested to see how well *T. couneifolia* extract and AgNPs treated groups affected their glucose tolerance levels. The results were compared to those of the standard medication DG group. In the DAgNPs-treated rats, blood glucose levels were significantly lower than in the control group. Over the course of two hours, the DG, DE, and DAgNPs groups had the most effective chemicals for reducing blood glucose levels. It has been found that D AgNPs therapy improves blood glucose levels that are equivalent to DC treatment. Several research have used the alloxan-induced diabetes rat model [59,60]. Furthermore, the mechanism of action of alloxan has been widely studied [61]. These findings are in line with other studies that have shown that the treated groups had a lower overall area under the curve than the DC group when using blood glucose concentration as a routine and significant biological measure to determine how well a patient’s condition is improving while receiving diabetes therapy [62].

Before and after treatment, the experimental rats’ body weights were compared. Swanston-Flatt et al. believe diabetes is related with weight loss, which may be attributed to muscle wastage [63]. Diabetes-induced rats (DC) demonstrated considerable weight loss in our experiment.

It is essential that lipid profile be performed in T2D patients since elevated lipid levels have been linked to cardiovascular infections and are typically detected in uncontrolled diabetes. Due to decreased cholesterol manufacturing in diabetes, cholesterol retention is reduced [64]. As a result, the enhanced activation of fatty acids from adipose tissue disrupts lipid metabolism. Hyperlipidemia was seen in healthy rats when alloxan was given to diabetic rats; this indicates that the medication causes hyperlipidemia in healthy rats [60]. Total cholesterol, triglycerides, HDL, and LDL levels were measured before and after treatment with *T. couneifolia* extract and AgNPs. A rise in cholesterol, triglyceride, and HDL levels was seen in alloxan-induced rats. To put it another way, alterations in serum lipid levels are unquestionably a consequence of unregulated production of lipids by prolonged CAMP [65]. In diabetic rats treated with AgNP, cholesterol, triglycerides, and LDL levels dropped significantly, whereas HDL levels rose [66].

Diabetes is a set of diseases that disturbs all the body organs. It was found that the liver effected due to this reason, the quantity of ALT, ALP, and SBR increased in diabetic animals (DAC) as compared to control animals, but AgNPs group had significantly the levels of ALT, ALP, and SBR levels [65].

Blood urea, albumin protein, and creatinine concentrations in diabetic animals (DAC) are higher than in the control group, which is related with diabetes. These findings are in line with previous works that AgNPs has reduced blood urea, albumin protein, and creatinine levels [66].

## 5. Conclusions

Plant biogenic silver nanoparticles using methanol extract of *T. couneifolia* as a reducing agent showed a significant green remedy for diabetes-associated syndromes as well as a potential anti-hyperlipidemic agent.

## Figures and Tables

**Table 1:** Qualitative phytochemical analysis of *T. couneifolia* methanolic extract.

Tests	Present (+)	Absent (-)
Coumarins		-
Terpenoids	+	
Amino acids	+	
Carbohydrates	+	
Sterols		-
Quinones	+	
Phenols	+	
Alkaloids	+	
Flavonoids	+	
Tannins	+	
Glycosides	+	
Proteins		-
Saponins		-

**Table 2:** Effect of methanolic extract and synthesized *T. couneifolia* AgNPs on urine analysis.

	LEU	URO	PRO	PH	SG	BIL	GLU
Group1–Control	-	-	-	5	1	-	-
Group2–DM	14	1.5	30 ± 0.3	9	1.55	1.09	255 ± 15
Group3–DM control	-	1	28 ± 0.4	4	1	1	-
Group4–T Extract	-	0.9	15	5.3	1.33	1.06	-
Group5–T NPs	-	1.1	14 ± 0.15	4.8	1.12	1.02	-

## Data Availability

As per Journal criteria.
